# Effects of Co-Varying Diel-Cycling Hypoxia and pH on Growth in the Juvenile Eastern Oyster, *Crassostrea virginica*

**DOI:** 10.1371/journal.pone.0161088

**Published:** 2016-08-22

**Authors:** Andrew G. Keppel, Denise L. Breitburg, Rebecca B. Burrell

**Affiliations:** 1 Department of Oceanography, United States Naval Academy, Annapolis, MD, United States of America; 2 Smithsonian Environmental Research Center, Edgewater, MD, United States of America; Auckland University of Technology, NEW ZEALAND

## Abstract

Shallow water provides important habitat for many species, but also exposes these organisms to daily fluctuations in dissolved oxygen (DO) and pH caused by cycles in the balance between photosynthesis and respiration that can contribute to repeated, brief periods of hypoxia and low pH (caused by elevated pCO_2_). The amplitude of these cycles, and the severity and duration of hypoxia and hypercapnia that result, can be increased by eutrophication, and are predicted to worsen with climate change. We conducted laboratory experiments to test the effects of both diel-cycling and constant low DO and pH (elevated pCO_2_) on growth of the juvenile eastern oyster (*Crassostrea virginica*), an economically and ecologically important estuarine species. Severe diel-cycling hypoxia (to 0.5 mg O_2_ L^-1^) reduced shell growth in juvenile oysters, as did constant hypoxia (1.2 and 2.0 mg O_2_ L^-1^), although effects varied among experiments, oyster ages, and exposure durations. Diel-cycling pH reduced growth only in experiments in which calcite saturation state cycled to ≤0.10 and only during the initial weeks of these experiments. In other cases, cycling pH sometimes led to increased growth rates. Comparisons of treatment effects across multiple weeks of exposure, and during a longer post-experiment field deployment, indicated that juvenile oysters can acclimate to, and in some cases compensate for initial reductions in growth. As a result, some ecosystem services dependent on juvenile oyster growth rates may be preserved even under severe cycling hypoxia and pH.

## Introduction

Environmental conditions fluctuate over a wide range of time scales and amplitudes. These fluctuations can expose organisms to periods of potentially harmful conditions lasting from moments to decades. In shallow waters, diel patterns of light penetration result in daily cycles of photosynthesis and thus diel patterns of oxygen production and carbon dioxide consumption. The temporal patterns of photosynthesis, in conjunction with oxygen consumption and carbon dioxide production by respiration, can result in daily periods of hypoxia (dissolved oxygen [DO] concentrations well below 100% saturation) and environmental hypercapnia (pCO_2_ above that in equilibrium with the atmosphere, resulting in low pH) and contrasting periods of high DO and pH [1]. Environmental factors such as wind, tide, and cloud cover can modify the amplitude and periodicity of these cycles. Repeated exposure to brief periods of hypoxia and hypercapnia may be harmful to estuarine organisms in spite of adaptations to the wide range of environmental conditions common in estuaries [2,3].

Eutrophication increases total biomass compared to non-eutrophic conditions, resulting in increased photosynthesis and respiration, and increased amplitude of diel-cycles [1,4,5]. At some shallow sites in the eutrophic Chesapeake Bay, for example, DO concentrations can range from near anoxia to well above 100% saturation and pH values can cycle one or more units on a daily basis [6,7]. Increasing atmospheric CO_2_ concentration also adds to acidification of aquatic environments [8,9], and through its effects on climate, is predicted to increase the severity and duration of hypoxic events [10].

Previous research on the effects of hypoxia and hypercapnia has primarily tested the effects of continuous exposure to one (hypoxia: e.g.,[11–13]; pH: e.g., [14,15]), or in some cases, both of these stressors (e.g.,[8,16]). For example, Gobler et al. [17] found additive and synergistic effects of continuous hypoxia and low pH on growth of larval scallops, *Argopecten irradians* (Lamarck, 1819). Hypoxia, but not acidification, reduced scallop growth; acidification, but not hypoxia, reduced survivorship; and there were interactive effects of DO and pH on metamorphosis. Fewer studies have investigated the effects of cycling hypoxia or hypercapnia [1,18], and replicating the two co-varying cycles has been rare (but see [19,20]). Because it is both common and likely to worsen, understanding the effect of co-varying hypoxia and acidification on shallow-water communities is vital to understanding the impact of eutrophication as well as predicting the consequences of climate change for ecologically and economically important estuarine systems.

Cycling conditions may have effects similar to those of continuous low dissolved oxygen and pH, or may affect organisms differently due to the rapid changes and frequent periods of respite interspersed among periods of potentially harmful conditions. Although mobile organisms will often relocate to avoid hypoxia exposure [21], Bell and Eggleston [22] found reduced avoidance behavior in blue crabs, *Callinectes sapidus* (Rathbun, 1896), exposed to sudden hypoxic events relative to those exposed to long-term hypoxia. In contrast, Taylor and Miller [23] found that southern flounder, *Paralichtys lethostigma* (Jordan and Gilbert, 1884), exposed to diel-cycling hypoxia experienced changes in hematocrit levels similar to those under constant hypoxia.

Diel-cycling DO and pH occur with other environmental conditions that may modulate not only the cycles themselves, but also the responses of estuarine organisms. Increased temperature and salinity decrease oxygen solubility [24], while warmer temperatures also increase the oxygen requirements of organisms [25]. The presence and proportion of fresh water mixing drive changes in salinity and alkalinity [26]. Low salinity and low alkalinity reduce the availability of calcite in aquatic systems [27]. Low salinity also reduces the assimilation rate of food in the Pacific oyster [28]. Conversely, increased food availability can allow organisms to withstand the increased energy demands associated with acidification [29] or hypoxia [30].

Estuarine organisms, which are adapted to live under a wide range of conditions, may be able to acclimate to, or compensate for, exposure to suboptimal conditions. Bogue [19] found that the mummichog, *Fundulus heteroclitus* (L. 1766), could acclimate to diel-cycling hypoxia after 10 days of exposure, such that growth was not affected in the second 10 day period. In contrast, Taylor and Miller [23] found that southern flounder, *Paralichthys lethostigma* (Jordan and Gilbert, 1884), acclimated to constant hypoxia exposure, but not to cycling hypoxia. Japanese ricefish, *Oryzias latipes* (Temminck and Schlegel 1846), compensated for initial developmental delays such that overall growth was not affected by constant hypercapnia [31]. Although previous research has demonstrated reduced growth in oysters under hypoxia (e.g., [32,33]) and hypercapnia (e.g., [14,34]), oysters have not previously been observed to acclimate to either stressor.

Calcifying organisms, such as oysters, are heavily dependent on the availability of calcium carbonate in the environment. The shells of juvenile and adult oysters are composed primarily of calcite rather than aragonite [35]. Calcification is more energetically costly at low calcium carbonate saturation states that occur in estuaries as a result of high respiration rates and the positive relationship between salinity and alkalinity. Calcium carbonate levels below saturation (Ω_calcite_ < 1.0) can result in the dissolution of carbonate compounds. Reduced calcite saturation resulting from elevated CO_2_ has been shown to reduce growth of eastern (*Crassostrea virginica* (Gmelin, 1791)) [14,15,36], Olympia (*Ostrea lurida* (Carpenter, 1864)) [37], and Pacific (*C*. *gigas* (Thunberg, 1793)) [34,38] oysters, and to increase mortality in eastern oysters [36].

The eastern oyster, is native to the western Atlantic from Brazil to Canada in waters with salinity above 5 PSU and temperature below 32°C; although it can survive brief periods of conditions exceeding these bounds [39,40]. Oysters couple the benthic and pelagic environments, filter the water column, engineer habitat [41], and support major fisheries through much of their range. Overfishing, habitat degradation, and disease have resulted in severe population declines. Stocks in Chesapeake Bay, for example, are estimated to be below 1% of historic levels [42,43]. As with many other sessile organisms, oysters tend to be tolerant of hypoxia [44,45], but chronic exposure to hypoxia has been shown to reduce feeding, metabolism, and growth [32,46,47]. Hypoxia can also result in adult oyster mortality and alter oyster reef community dynamics [48,49]. Exposure to severe diel-cycling hypoxia increases infection acquisition and progression [6,50] and exposure to cycling hypoxia or cycling pH, as well as both cycles in conjunction, stimulate immune activity [50].

This research examined the effects of diel-cycling DO and co-varying pH, as well as each stressor individually and under constant conditions, on growth of juvenile *C*. *virginica* (Gmelin, 1791). Although there is a plethora of DO and pH data available for shallow water environments in Chesapeake Bay [7], other carbonate chemistry parameters for these sites are not well measured. For this reason, we designed our experiments around pH targets although we assume that the availability of calcium carbonate is the primary driving force behind pH effects on oyster growth seen here.

## Methods

**Fig 1:**
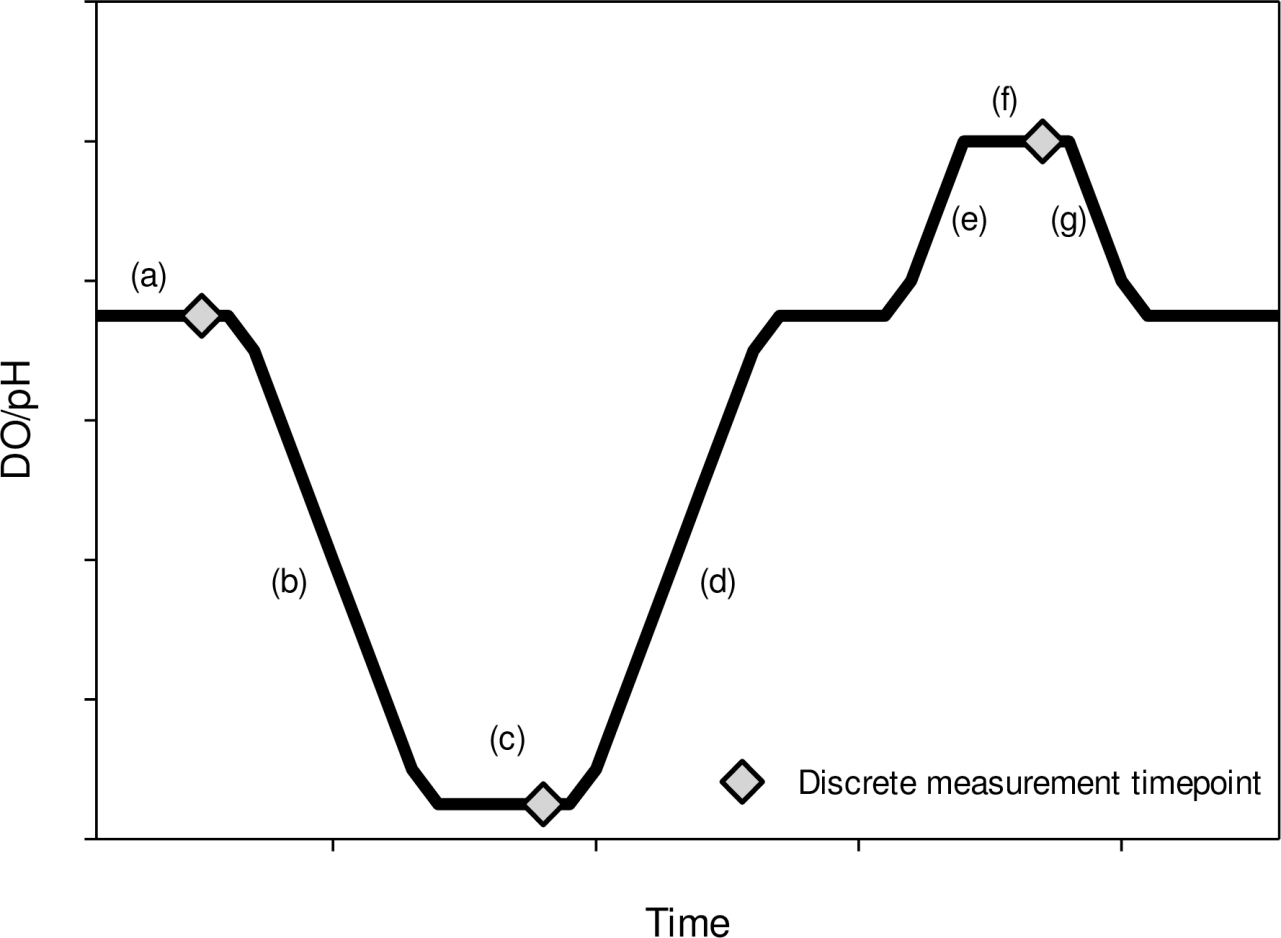
Idealized diel cycle for laboratory experiments after Burrell et al. (2015). Cycles are designated as: (a) ‘normoxia’, (b) ‘down-to-low’, simulated late evening, (c) ‘low plateau’–which can refer to low DO, low pH or both, simulated pre- dawn, (d) ‘up-to-normoxia’, simulated dawn, (e) ‘up-to-supersat’, simulated early afternoon, (f) ‘supersat plateau’, simulated midafternoon, (g) ‘down-to-normoxia’, simulated late afternoon. Diamond markers indicate time points where discrete measurements of DO and pH were taken in each aquarium.

We tested the effects of diel-cycling DO and pH on growth of juvenile eastern oysters in four experiments conducted from 2012 to 2015 at the Smithsonian Environmental Research Center (SERC), in Edgewater, Maryland, USA. Fig 1 depicts a stylized daily cycle and names used for the various phases of the cycles. Treatment names and DO and pH mean values measured at various parts of the cycles are provided in Table 1.

**Table 1:** Mean ± SE (n) DO and pH in oyster growth experiments.

Treatment	DO & pH	2012	2013	2014	2015
Normoxia,	HDO:	7.31 ± 0.02 (259)	7.80 ± 0.02 (10)	7.82 ± 0.05 (12)	7.36 ± 0.02 (42)
Normocapnia	LDO:	7.42 ± 0.03 (236)	7.97 ± 0.03 (85)	7.88 ± 0.02 (121)	7.33 ± 0.04 (144)
(Control)	HpH:	7.83 ± 0.00 (257)	8.09 ± 0.01 (120)	7.96 ± 0.01 (152)	8.01 ± 0.01 (42)
	LpH:	7.84 ± 0.01 (242)	8.12 ± 0.00 (85)	7.98 ± 0.01 (128)	7.88 ± 0.01 (144)
Normoxia, cycling	HDO:	7.31 ± 0.02 (258)	7.78 ± 0.02 (10)	7.84 ± 0.06 (19)	7.41 ± 0.05 (36)
pH	LDO:	7.37 ± 0.03 (238)	7.92 ± 0.03 (85)	7.88 ± 0.02 (119)	7.27 ± 0.03 (138)
	HpH:	7.82 ± 0.00 (257)	8.03 ± 0.00 (120)	7.92 ± 0.01 (151)	7.93 ± 0.01 (36)
	LpH:	6.99 ± 0.00 (244)	7.00 ± 0.01 (85)	7.11 ± 0.00 (128)	6.70 ± 0.00 (138)
Moderate cycling	HDO:		7.78 ± 0.02 (10)		
hypoxia,	LDO:		1.31 ± 0.01 (84)		
Normocapnia	HpH:		8.04 ± 0.01 (120)		
	LpH:		8.10 ± 0.01 (85)		
Moderate cycling	HDO:	7.30 ± 0.02 (259)			
hypoxia, cycling	LDO:	1.71 ± **0**.01 (237)			
pH	HpH:	7.81 ± 0.00 (257)			
	LpH:	7.04 ± 0.01 (243)			
Severe cycling		7.26 ± 0.02 (259)	7.77 ± 0.02 (10)	7.84 ± 0.04 (21)	7.35 ± 0.02 (42)
hypoxia,		0.59 ± 0.01 (236)	0.55 ± 0.00 (84)	0.51 ± 0.01 (128)	0.57 ± 0.01 (138)
Normocapnia		7.81 ± 0.00 (257)	8.03 ± 0.01 (120)	7.93 ± 0.01 (152)	7.90 ± 0.03 (42)
		7.84 ± 0.00 (242)	8.08 ± 0.01 (85)	8.05 ± 0.01 (128)	7.86 ± 0.01 (138)
Severe cycling	HDO:			7.84 ± 0.05 (21)	
hypoxia, Moderate	LDO:			0.53 ± 0.01 (128)	
cycling pH	HpH:			7.91 ± 0.01 (152)	
	LpH:			7.46 ± 0.00 (128)	
Severe cycling	HDO:	7.32 ± 0.02 (259)	7.76 ± 0.02 (10)	7.83 ± 0.05 (22)	7.31 ± 0.02 (42)
hypoxia, cycling	LDO:	0.57 ± 0.01 (238)	0.56 ± **0**.01 (85)	0.52 ± 0.01 (128)	0.63 ± 0.01 (132)
pH	HpH:	7.84 ± 0.00 (257)	8.03 ± 0.00 (120)	7.91 ± 0.01 (151)	7.91 ± 0.01 (42)
	LpH:	7.02 ± 0.00 (244)	6.99 ± 0.00 (85)	7.09 ± 0.00 (126)	6.71 ± 0.01 (132)
Normoxia,	HDO:		7.79 ± 0.01 (10)		
Constant moderate	LDO:		7.97 ± 0.03 (85)		
pH	HpH:		7.41 ± 0.01 (119)		
	LpH:		7.35 ± 0.01 (85)		
Constant	HDO:		1.28 ± 0.01 (85)	2.07 ± 0.01 (22)	
moderate/mild	LDO:		1.28 ± 0.01 (130)	2.09 ± 0.01 (128)	
hypoxia,	HpH:		8.05 ± 0.01 (85)	8.02 ± 0.01 (152)	
Normocapnia	LpH:		8.05 ± 0.00 (199)	8.03 ± 0.01 (128)	

Mean ± SE (n) DO and pH in oyster growth experiments on days on which treatment conditions cycled. High dissolved oxygen, high pH (HDO, HpH): DO and pH measured in aquaria at simulated late afternoon portion of the daily cycle when pH and DO were at or near their daily maxima in cycling treatments (i.e. high). Low dissolved oxygen, low pH (LDO, LpH): DO and pH measured in aquaria at simulated dawn when pH and DO were at their daily minima in cycling treatments (i.e. low). Empty cells are treatments which were not performed during the experiment in that column.

Experimental treatments varied among years in order to test for repeatability of results and to follow up on results of early experiments. Briefly, the 2012 experiment consisted of one control (both DO and pCO_2_ near air-saturated levels throughout the 24-h cycle) and four cycling treatments, but no constant hypoxia or constant reduced pH (hypercapnia) treatment. In the 2013 experiment, two additional treatments, constant moderate hypercapnia and constant moderate hypoxia, were added to tease apart effects of cycling versus constant hypercapnia and hypoxia. Additionally, the moderate cycling hypoxia treatment was adjusted from 1.5 mg L^-1^ to 1.3 mg L^-1^ in an effort to identify a DO threshold at which effects might occur; additionally, this moderate cycling DO treatment was run without co-varying pH. For the 2014 experiment, the target DO level of the constant hypoxia treatment was increased from 1.3 mg L^-1^ to 2.0 mg L^-1^, to attempt to identify the upper DO concentrations at which growth might be reduced. In addition, all treatments were run at both ambient and supplemented chlorophyll levels to test for interactive effects of food availability and DO or pH. In the final experiment, conducted in 2015, four treatments were run using the original factorial design crossing cycling hypoxia with cycling hypercapnia. The DO target for this experiment remained 0.5 mg L^-1^, but the pH target was changed to 6.7 to include a treatment representative of pH measurements made in a local saltmarsh creek [51].

Eyed oyster larvae produced from wild-caught Chesapeake Bay broodstock were obtained from the Horn Point Oyster Hatchery (Cambridge, MD, USA). Although not collected from the wild, larval oysters were included on a scientific collection permit approved by the Maryland Department of Natural Resources. Larvae were placed in 0.25 m^3^ raceways at SERC with roughened 12.7 × 12.7 × 0.5 cm poly-vinyl chloride (PVC) (2012) or acrylonitrile butadiene styrene (ABS) (2013–2015) tiles in 0.54 μm filtered Rhode River water modified when necessary using Coralife Scientific Grade Marine Salt (Coralife, Central Aquatics, Franklin, WI, USA) to match the salinity at which larvae had been hatched (~10 PSU). After three days, raceways were put on flow-through 0.54 μm filtered Rhode River water, and fed intermittently with stock algal diet (DT’s Reef Blend, http://www.dtplankton.com). One age class of larvae was used in the 2012, 2014, and 2015 experiments and three age classes were used in the 2013 experiment. Larvae were set four weeks prior to the experiment’s start in 2012, four weeks, two weeks, and one week prior to the experiment in 2013, three weeks prior to the start of the 2014 experiment, and four weeks prior to the start of the 2015 experiment.

Tiles were removed from settlement raceways, photographed, and 1–3 tiles per age class were placed into six replicate 75 L experimental aquaria at the start of each treatment (July 25, 2012, August 29, 2013, May 29, 2014, and July 2, 2015). Each aquarium received 12 juvenile oysters in 2012, 5–8 juveniles per age class in 2013, 5–8 juveniles in 2014, and 10–22 juveniles in 2015. Variation in numbers among years reflected variation in numbers of juveniles that successfully settled onto tiles and the desire to avoid oysters growing onto each other during the experiment. Multiple tiles were used in cases where settlement was not dense enough to achieve target numbers of individuals per tank with single tiles. In cases of large variation in oyster numbers (first two settlements in 2013, all oysters in 2015), replicates were blocked by number of oysters per tank. In 2013, the three age classes of juvenile oysters were placed together in the same experimental aquaria.

Tiles were oriented vertically several centimeters above the bottom of the tanks in order to avoid buildup of sediment on top of oysters. Incandescent 5 V rope-lighting was used to replicate light levels at a depth of 2 m in the Rhode River on a sunny day. Photoperiod regime was maintained in a 14 hours light:10 hours dark cycle seven days per week. In all experiments, a randomized block design was used clustering one replicate from each treatment together to account for any environmental gradients associated with laboratory position. After the initial settlement period, juvenile oysters were moved to experimental aquaria and allowed to acclimate to water flow, light levels, and feeding regimes for four days at normoxia and normocapnia before experimental treatments started. Photographs and image analysis software (ImageJ, v. 1.37, National Institutes of Health, USA) were used to measure juvenile oyster areas. In some cases (youngest age class in 2013 and all individuals in 2014), oysters were too small initially to be efficiently measured by photography. In these cases, a subset was measured using a microscope and stage micrometer and found to be ≤1 mm^2^.

DO and pH conditions were controlled by a custom-developed LabVIEW-based (National Instruments Corp., Austin, TX, USA) diel-cycling laboratory system [51]. Each aquarium containing juvenile oysters was bubbled with a constant volume of gas comprised of up to five different gasses, including N_2_, CO_2_, O_2_, and either atmospheric or CO_2_-stripped air. The proportion of each gas in the mix was calculated by the LabVIEW program based on designated DO and pH targets and feedback from sensors, and was regulated with mass flow controllers (Dakota Instruments, Orangeburg, NY, USA). One gas mix was created for each treatment and then split equally among replicates. DO and pH were monitored in one replicate of each treatment using Oxyguard Standard DO probes (Oxyguard International A/S, Birkeroed, Denmark) and Honeywell Durafet III pH sensors (Honeywell International, Morristown, NJ, USA). Because the LabVIEW program only had the ability to monitor and control five treatments, non-cycling treatments in some experiments were created externally from the LabVIEW program using manual flow meters and Saga pH-2002C Digital pH-ORP Controllers (Saga Electronic Enterprise Co., Ltd., New Taipei City, Taiwan).

Treatments were cycled 4–7 days per week for seven weeks (2012), five weeks (2013), two weeks (2014), or five weeks (2015). The 2014 experiment was originally designed to last five weeks (like the 2013 and 2015 experiments) but was cut short because of a problem with our supplemental algae. Constant treatments were maintained continuously for the length of the experiments. On days when conditions did not cycle, all treatments were bubbled with air and CO_2_-stripped air to maintain DO and pH values similar to the control treatment. In the field, environmental conditions (wind, temperature, solar irradiance, etc.) can result in days on which temporal variation in DO and pH is small [1] and both DO and pH remain well above levels previously shown to affect oysters [6,52]. Normocapnia, was operationally defined as pCO_2_ levels resulting in a pH between 7.8 and 8.1 –pH levels achieved in our system by bubbling raw estuarine water with CO_2_-stripped air. To determine whether all replicates were similar to those monitored and controlled by the LabVIEW system, discrete measures of DO, temperature, and salinity were made three to four times per day (Fig 1) in all replicate aquaria using a YSI ProfessionalPlus meter (Yellow Springs Instruments, Yellow Springs, OH, USA) and pH was measured at the same times using an Oakton Acorn pH 5 meter (Oakton Instruments, Vernon Hills, IL, USA). In 2015, an Orion Star A326 (Thermo Fischer Scientific, Waltham, MA., USA) portable meter was used in place of the YSI and Oakton meters.

In 2012 only, juvenile oysters were raised in aquaria that also contained one year-old oysters being monitored for Dermo acquisition and progression [50]. Each aquarium received 1 L min^-1^ of flow-through, unfiltered, Rhode River water supplemented with 0.093 mL of stock algal diet (DT’s Reef Blend, http://www.dtplankton.com/) mixed into the inflow water every eight minutes, 24 hours per day, throughout the experiment, with the exception of a 10 day period in August during which the timer controlling the algae system was under repair. Each aquarium also received 75 mL min^-1^ of water from a holding tank containing approximately 400 adult oysters infected with *Perkinsus marinus* (Levine, 1978), the protistan parasite that causes Dermo disease in oysters. Juveniles do not develop detectable infections of *P*. *marinus* and their growth should not have been negatively affected by presence of the pathogen [53]. However, because one year-old oysters would have competed for available phytoplankton, and experimental treatments affected adult filtration rates, food availability likely varied among treatments. Severe hypoxia depresses adult feeding rates but results in compensatory feeding during high oxygen phases of the daily cycle; low pH in the absence of co-occurring, low DO can also stimulate filtration slightly [6,52,54].

In 2013 each aquarium received 0.3 L min^-1^ of Rhode River water supplemented with continuous additions of 0.088 mL min^-1^ of stock algal diet. In 2014, each aquarium received 0.3 L min^-1^ of Rhode River water and half of the aquaria received supplemental algae. Aquaria in the supplemented food treatment received continuous additions of 0.11 mL min^-1^ of stock algal diet while the non-supplemented treatment only received water from the SERC river water system. In 2015, oyster tanks received 0.5 L min^-1^ of Rhode River water and no additional dietary supplementation; the higher flow rate was intended to at least partially compensate for lack of supplemental feeding. Variation among years in supplemental feeding reflected our attempts to compensate for the difference in chlorophyll *a* levels between the Rhode River and the water delivered to our laboratory tanks. Salinity and alkalinity were allowed to vary with ambient conditions in the Rhode River, as was temperature in 2012, 2013, and 2015. Due to the earlier experimental dates in 2014, incoming water was warmed that year to keep temperatures close to those of previous experiments.

All tiles from the 2012 and 2013 juvenile oyster experiments were photographed after the period of acclimation and before treatment cycles commenced, again at the mid- (August 7 and August 27, 2012, September 17, 2013) and end-points (October 3, 2012, October 8, 2013) of each experiment. Oysters from the 2014 experiment were only analyzed after two weeks of exposure to treatments (June 10, 2014). Oysters from the 2015 experiment were photographed weekly for the duration of the five week experiment to more closely examine temporal patterns of treatment effects. Two weeks into the 2012 experiment, oysters were thinned haphazardly to six individuals per replicate aquarium to avoid overcrowding on tiles. This was also done in the youngest age class of the 2013 experiment after two weeks. All photographs were processed using image analysis software with the same methods as those at the start of experiments. Any mortality was noted at the end-point of each experiment.

After the endpoint sampling in 2012, tiles from each aquarium were deployed in the Rhode River. Oysters were deployed with approximately 2 m spacing between replicates, hanging from piers 0.5 m above the bottom in approximately 2 m of water to avoid periods of air exposure. Three sites were necessary to find enough dock area to space out oysters. Field conditions are certainly more variable than treatments under laboratory control; however previous data suggest that dissolved oxygen conditions were less severe than any of our laboratory cycling treatments [6], and all treatments would have been exposed equally to field conditions. Laboratory blocks were maintained in the field, with two replicates going to each of two sites, and the fifth replicate deployed at the third site. Juvenile oysters were retrieved and re-measured in July of 2013, after nine months of field deployment.

### Other Measurements

Alkalinity was measured thrice weekly during all experiments to calculate calcite saturation states using CO_2_SYS.XLS [55]. Samples were filtered to 0.45 μm and kept at 4°C until processing. In 2012 alkalinity samples were processed according to Standard Methods 2320 [56], and in 2013–2015 according to the Guide to Best Practices for Ocean CO_2_ Measurements [57] and using certified reference material from the Dickson lab at Scripps Institution of Oceanography.

### Statistics

Shell areas of juvenile oysters were used to calculate instantaneous growth rates. In cases where initial measurements could not be made (youngest age class of oysters in 2013 and all oysters in 2014), starting size was assumed to be 1 mm^2^ in calculations. Statistics were performed on means within aquaria. Unless otherwise noted, data are presented as means ± one standard error. Any differences referred to as significant are significant at p = 0.05. Some non-significant trends (0.05 < p < 0.10) are also discussed.

All data were tested for homogeneity of variance using an F-max test and normality using a Shapiro-Wilkes test. All statistical analyses were performed in the Proc Mixed procedure in SAS (SAS Institute Inc., Cary, NC, USA). Effects of cycling treatments on mortality were examined for every experiment using a randomized complete block design (RCBD) ANOVA with laboratory position as the blocking factor. Growth rates during each portion of the experiments, as well as oyster size at the end of experiments, were analyzed to examine differences in growth rates among treatments during different time periods that might indicate acclimation to- or compensation for- exposure to experimental conditions. Effects of DO and pH on growth rates during the laboratory experiments were analyzed as RCBD ANCOVAs with laboratory position as the blocking factor and size at the start of the time interval of interest as the covariate. In cases where individual starting sizes were not available (e.g., youngest age class of 2013 experiment and all oysters in 2014 experiment) mid-point growth rates were analyzed as RCBD ANOVAs. Growth rates during the field recovery portion of the 2012 experiment were also analyzed with an ANCOVA using juvenile oyster shell area at the time of deployment as the covariate. Shell areas were analyzed as RCBD ANCOVAs with initial shell area as the covariate (when available) and laboratory position as the blocking factor. For the 2014 juvenile oyster growth experiment, results were first analyzed using a two-way ANOVA testing for an interactive effect of food treatment with DO/pH treatment.

Least square means contrasts were used to test for interactive effects of severe cycling DO and cycling pH as well as *a priori* hypotheses that cycling DO and cycling pH would reduce growth relative to controls. *A priori* hypotheses that constant low DO and pH would reduce growth as compared to the controls and that constant conditions would not differ from similar cycling conditions were also tested using least square means comparisons. Pre-planned comparisons were performed regardless of overall test significance [58].

## Results

### Overview

Juvenile oysters grew substantially under laboratory conditions (Table 2) and exhibited a variety of responses to cycling DO and pH both among experiments and among time periods within experiments (summarized in Table 3). Neither diel-cycling DO nor diel-cycling pH consistently decreased growth throughout the entire duration of experiments even though daily minimum conditions to which oysters were exposed were as severe as 0.5 mg L^-1^ DO and 6.70 pH (Tables 1 and 3). In several cases, results indicated that juvenile oysters may acclimate to, or compensate for exposure to cycling conditions (Table 3 and below). Juvenile oyster mortality ranged from 0.0 to 18.0% in the various treatment*experiment combinations. There were no differences in mortality among treatments within any of the laboratory experiments (Table 4).

**Table 2:** Starting, ending, and recovery shell area of oysters.

Experiment year/cohort	Starting	~2 weeks	~4–5 weeks	7 weeks	Recovery
2012	52.6 ± 0.1, 30,	210.6 ± 8.2, 30,	430.1 ± 19.3, 30,	486.9 ± 19.0, 30,	1345.2 ± 47.9, 27,
	(1.2–2.8)	(81.6–286.8)	(182.9–612.4)	(298.1–683.1)	(706.8–1720.7)
2013–4 weeks post-settlement cohort	17.2 ± 1.9, 35,	248.3 ± 14.4, 35,	537.1 ± 31.3, 34,		
	(3.7–59.8)	(58.7–457.5)	(104.5960.7)		
2013–2 weeks post-settlement cohort	3.6 ± 0.1, 35,	153.4 ± 9.3, 35,	437.1 ± 21.6, 34,		
	(1.8–5.4)	(38.9–291.7)	(120.0–731.7)		
2013–1 week post- settlement cohort	≤1	95.9 ± 5.01, 35,	340.4 ± 18.8, 34,		
		(37.0–142.6)	(131.4–558.0)		
2014	≤1	12.1 ± 0.3, 48,			
		(8.7–18.1)			
2015	10.1 ± 0.6, 24,	71.9 ± 4.8, 24,	291.5 ± 13.5, 24,		
	(5.3–18.7)	(29.4–124.2)	(194.0–435.9)		

Starting, ending, and recovery shell area (mm^2^) of oysters for each experiment, mean ± SE, sample size, and range (in parenthesis). Midpoint, endpoint, and recovery means are means of all replicates of all treatments. Empty cells are time points not measured in that experiment.

**Table 3:** Summary of Ω_calcite_ and growth results from 2012 through 2015 experiments.

Calcite saturation states and effects of oxygen and pH treatments on oyster growth rates	2012	2013–4 weeks post settlement cohort	2013–2 weeks post settlement cohort	2013–1 week post settlement cohort	2014	2015
Ambient calcite saturation state	1.05	1.87	1.87	1.87	0.69	1.01
Low pH calcite saturation state	0.19	0.18	0.18	0.18	0.10	0.05
Severe cycling hypoxia–first part of experiment	**↓**	↔	**↓**	**(↓)**	**↓**	**↓**
Severe cycling hypoxia–second part or after experiment	**↑**	↔	↔	↔		**↑**
Constant hypoxia–first part of experiment		**↓**	**↓**	**↓**	**↓**	
Constant hypoxia–second part of experiment		**↓**	↔	↔		
Cycling pH–first part of experiment	↔	↔	↔	↔	**↓**under normoxia	**↓**under normoxia and hypoxia
Cycling pH–second part of experiment	**↑** under severe cycling hypoxia	↔	↔	↔		**↑** under normoxia
Constant hypercapnia		↔	↔	↔		
Acclimation/compensation (early negative effect of hypoxia or low pH ceased or reversed later in experiment)	√		√	√		√

Summary of Ω_calcite_ and growth results from 2012 through 2015 experiments. Ambient calcite saturation states were measured in the pH control treatments; Low pH calcite saturation states were measured in the cycling treatment during the low pH/low DO phase of the cycle. Upward and downward arrows without brackets indicate significant (p < 0.05) positive or negative effects of treatments, respectively. Arrows and in brackets indicate 0.05 < p < 0.1. Sideways arrows indicate no significant effect of treatment. Empty cells indicate effect was not examined in that experiment. All comparisons are relative to control conditions for that parameter.

**Table 4:** Mean tank mortality during each of the growth experiments.

	df	F	P
2012	4, 20	0.82	0.529
2013–4 weeks post settlement	6, 24	0.24	0.959
2013–2 weeks post settlement	6, 24	0.61	0.719
2013–1 weeks post settlement	6, 24	1.30	0.295
2014	11, 33	1.03	0.441
2015	3, 15	0.98	0.426

Randomized complete block design ANOVA of mean tank mortality during each of the growth experiments. ANOVA source and factor was treatment for all experiments. No significant effects were found.

Conducting experiments over the course of four summers resulted in a range of environmental conditions (i.e. Ωcalcite, chlorophyll *a*, salinity, and temperature). Dates of experiments and water quality parameters common to all experimental treatments are presented in Table 5. Temperature during the 2012 experiment was warmer than during the other three experiments, but all experiments were conducted at temperatures that occur within the natural range of the eastern oyster [40].

**Table 5:** Experimental dates, mean ± SE and range of water quality parameters in treatment aquaria during growth experiments 2012–2015.

	2012	2013	2014	2015
Dates	7/25/2012–	8/29/2013–	5/29/2014–	7/2/2015–
	10/3/2012	10/8/2013	6/27/2014	8/10/2015
Salinity (PSU)	10.66 ± 0.00	11.90 ± 0.01	6.10 ± 0.01	8.36 ± 0.01
	(9.25–12.28)	(9.2–12.93)	(5.36–6.81)	(7.22–9.47)
Temperature	29.49 ± 0.01	24.55 ± 0.02	25.08 ± 0.02	27.20 ± 0.03
(°C)	(24.83–31.36)	(21.97–27.03)	(22.88–27.17)	(23.9–29.2)
Total Alkalinity	1614.7 ± 15.8	1678.9 ± 4.97	1174.3 ± 16.03	1382.64
(μmol kg^-1^ sw)	(1524.0–1700.7)	(1664.6–1692.9)	(1089.45–1273.31)	(1292.09–1505.61)
Chl *a* (μg L^-1^)			Algae added:	
			4.272 ± 0.185	
		4.075 ± 0.137	(0.722–13.422)	5.339 ± 0.103
		(1.343–9.869)	Ambient:	(2.853–8.004)
			2.829 ± 0.116	
			(0.899–11.290)	

Experimental dates, mean ± SE and range of water quality parameters in treatment aquaria during growth experiments 2012–2015. Chl *a* is the concentration of chlorophyll *a* in the water column as measured by fluorescence. Empty boxes are variables not measured during that experiment.

### Juvenile growth

**Fig 2:**
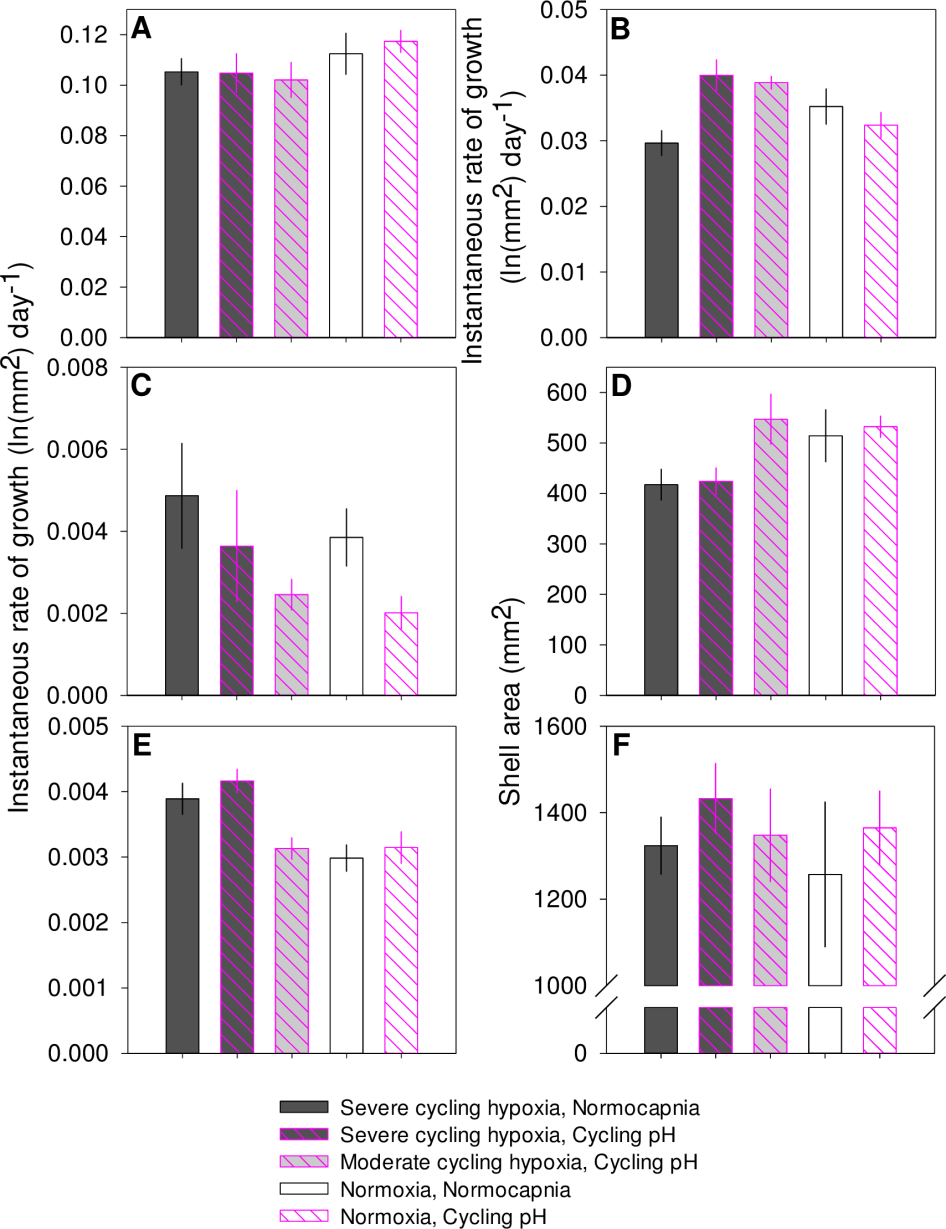
2012 juvenile growth experiment. Mean ± SE instantaneous rate of growth in area by treatment of juvenile oysters exposed to diel cycles 4–5 d wk^-1^ during (A) the first two weeks, (B) second two weeks, and (C) weeks four-seven. (D) shell area at the conclusion of the laboratory growth experiment. (E) instantaneous rate of growth during a 9-month field deployment and (F) shell area at the conclusion of the 9-month field deployment.

In 2012, juvenile oysters four weeks post-settlement at the start of the experiment grew an average of 439.4 ± 18.9 mm^2^ (n = 30 tanks) during the laboratory exposure phase of the study. Juvenile oysters exposed either to severe or moderate cycling hypoxia had significantly lower rates of growth in shell area than normoxic/normocapnic controls during the first two weeks of exposure (Table 6A, Fig 2A). The effect of cycling hypoxia was small, however, with only a 7% reduction in growth rates relative to controls even in the severe cycling hypoxia treatment. Cycling pH did not affect oyster growth rates during this portion of the experiment.

**Table 6:** 2012 juvenile growth experiment.

**A. 2 weeks, Instantaneous Growth**			
ANCOVA Source and Factor	df	F	P
Starting Shell Area	1, 9.73	1.84	0.206
Treatment	4, 22.06	2.67	0.059
Contrasts	df	t	P
Severe cycling hypoxia*Cycling pH Interaction	19	0.87	0.398
Severe cycling hypoxia vs. Normoxia	19	3.77	**0.001**
Severe cycling hypoxia vs. Moderate cycling hypoxia	19	0.90	0.379
Moderate cycling hypoxia vs. Normoxia	19	2.50	**0.022**
Cycling pH vs. Normocapnia	19	0.51	0.616
**B. 2–4 weeks, Instantaneous Growth**			
ANOVA Source and Factor	df	F	P
Two Week Shell Area	1, 8.304	0.29	0.606
Treatment	4, 23.843	5.01	**0.005**
Contrasts	df	t	P
Severe cycling hypoxia*Cycling pH Interaction	19	2.72	**0.014**
Severe cycling hypoxia vs. Normoxia under Normocapnia	19	2.01	0.059
Severe cycling hypoxia vs. Normoxia under Cycling pH	19	2.26	**0.036**
Severe cycling hypoxia vs. Moderate cycling hypoxia	19	0.39	0.701
Moderate cycling hypoxia vs. Normoxia	19	2.43	**0.026**
Cycling pH vs. Normocapnia under Normoxia	19	1.10	0.284
Cycling pH vs. Normocapnia under Hypoxia	19	3.95	**0.001**
**C. 4–7 week, Instantaneous Growth**			
ANOVA Source and Factor	df	F	p
Four Week Shell Area	1, 18.038	40.14	**<0.001**
Treatment	4, 20.628	1.40	0.268
Contrasts	df	t	P
Severe cycling hypoxia*Cycling pH Interaction	19	1.40	0.177
Severe cycling hypoxia vs. Normoxia	19	0.90	0.380
Severe cycling hypoxia vs. Moderate cycling hypoxia	19	0.51	0.616
Moderate cycling hypoxia vs. Normoxia	19	0.26	0.796
Cycling pH vs. Normocapnia	19	2.02	0.057
**D. Endpoint Area**			
ANOVA Source and Factor	df	F	p
Starting Shell Area	1, 12.173	3.93	0.070
Treatment	4,21.181	2.35	0.087
Contrast	df	t	P
Severe cycling hypoxia*Cycling pH Interaction	19	0.27	0.790
Severe cycling hypoxia vs. Normoxia	19	2.68	**0.015**
Severe cycling hypoxia vs. Moderate cycling hypoxia	19	2.91	**0.009**
Moderate cycling hypoxia vs. Normoxia	19	0.63	0.538
Cycling pH vs. Normocapnia	19	0.46	0.653
**E. Recovery Instantaneous growth**			
ANOVA Source and Factor	df	F	P
Deployment Shell Area	1, 13.525	12.85	**0.003**
Treatment	4, 19.244	4.22	**0.013**
Contrasts	df	t	P
Severe cycling hypoxia*Cycling pH Interaction	16	0.09	0.932
Severe cycling hypoxia vs. Normoxia	16	3.39	**0.004**
Severe cycling hypoxia vs. Moderate cycling hypoxia	16	2.48	**0.025**
Moderate cycling hypoxia vs. Normoxia	16	0.33	0.747
Cycling pH vs. Normocapnia	16	1.32	0.205
**F. Recovery Area**			
ANOVA Source and Factor	df	F	P
Starting Shell Area	1, 9.729	0.28	0.611
Treatment	4, 20.179	0.84	0.517
Contrast	df	T	P
Severe cycling hypoxia*Cycling pH Interaction	16	0.30	0.771
Severe cycling hypoxia vs. Normoxia	16	0.75	0.463
Severe cycling hypoxia vs. Moderate cycling hypoxia	16	0.53	0.606
Moderate cycling hypoxia vs. Normoxia	16	0.09	0.926
Cycling pH vs. Normocapnia	16	1.44	0.171

Randomized complete block design ANCOVA of instantaneous rate of growth in shell area during (A) first two weeks, (B) second two weeks, and (C) weeks four through seven of experiment using size at the start of each time interval as a covariate. (D) Randomized complete block design ANCOVA of shell area at the end of the laboratory treatment exposure using starting shell size as a covariate. (E) RCBD ANCOVA of instantaneous rate of growth in shell area during field recovery using shell area at deployment as a covariate and (F) randomized complete block design ANCOVA of mean tank juvenile oysters area at the end of the recovery period (post-nine month field deployment) with lab placement as the blocking factor and deployment area as the covariate. Tests are considered significant at *a* = 0.05 and significant p values are bolded.

Instantaneous growth rates during the second two weeks of the 2012 experiment were lower than during the first two weeks and there was a significant interactive effect of cycling hypoxia and pH. Oysters grown under either severe or moderate diel-cycling hypoxia along with co-varying pH exhibited compensatory growth; growth rates of these oysters were significantly higher than growth rates of control oysters held under normoxia (Table 6B, Fig 2B). In contrast, there was a trend toward reduced growth rates under severe cycling hypoxia and normocapnic conditions compared to the control treatment. Cycling pH under hypoxia significantly stimulated growth rates compared to non-cycling pH under hypoxia. In the third interval of the experiment, cycling hypoxia had no effects on growth rate; but, there was a trend toward slower growth under cycling pH than under normocapnic conditions (Table 6C, Fig 2C). These variable treatment effects on growth over the course of the experiment resulted in smaller oysters in the severe cycling hypoxia treatments than in either the normoxia or moderate cycling hypoxia treatments at the end of the laboratory exposure, but no differences in size between cycling pH and normocapnia treatments (Table 6D. Fig 2D).

During the nine month field deployment in the Rhode River, oysters added an additional 865.3 ± 38.1 mm^2^ (n = 27) of shell area on average. Oyster survival among experimental units ranged from 17 to 100%, but did not vary among treatments. Mean oyster shell area at the end of the recovery period was 1345.2 ± 47. 9 mm^2^ (n = 27) and shell areas were similar regardless of prior exposure to pH or DO treatments during the laboratory experiment (Tables 2F and 6F). Oysters that were previously exposed to severe hypoxia grew significantly faster during the field deployment than oysters that had experienced constant normoxia or moderate cycling hypoxia (Table 6E, Fig 2E). Cycling pH did not have any significant latent effects on growth rates during this period (Table 6E).

**Fig 3:**
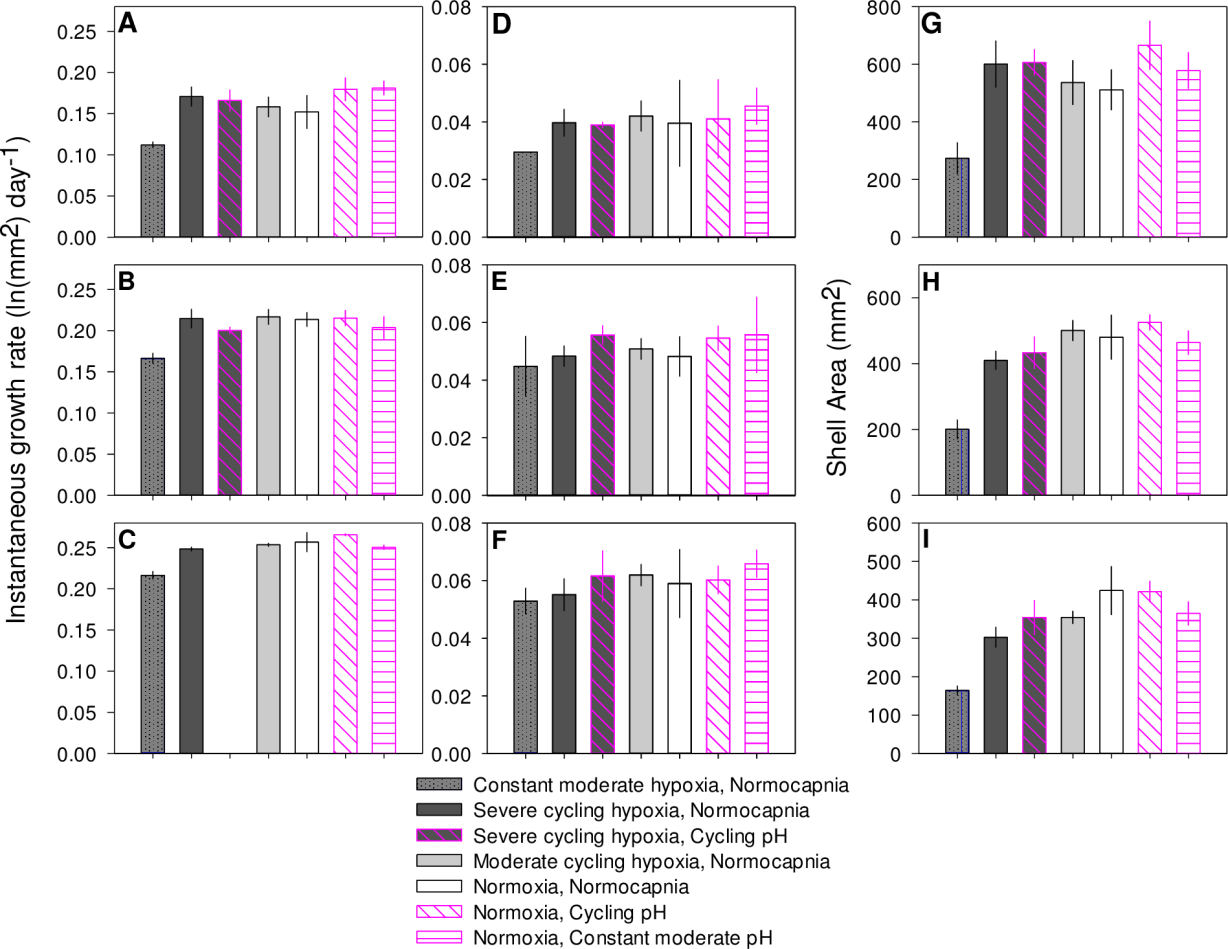
2013 juvenile growth experiment. Mean ± 1 SE instantaneous rate of growth in shell area during first two weeks of the experiment for three age classes of juvenile oysters; (A) 4 weeks post-settlement cohort, (B) 2 weeks post-settlement cohort, and (C) 1 week post-settlement cohort. Mean ± 1 SE instantaneous rate of growth in shell area during second two weeks of the experiment for three age classes of juvenile oysters; (D) 4 weeks post-settlement cohort, (E) 2 weeks post-settlement cohort, and (F) 1 week post-settlement cohort. Mean ± 1 SE shell area of (G) 4 weeks post-settlement cohort, (H) 2 weeks post-settlement cohort, and (I) 1 week post-settlement cohort of juvenile oysters at the end of the month-long experimental exposure.

In 2013, three separate age classes of juvenile oysters, those settled four weeks, two weeks, and one week prior to the start of experiments, were grown under experimental conditions. The oldest cohort of juvenile oysters grew an average of 521.1 ± 31.9 mm^2^ (n = 34), and instantaneous growth rates were not significantly affected by any cycling treatment during any time period evaluated, nor were ending shell areas (Table 7A, 7D and 7G, Fig 3A, 3D and 3G). Oysters settled two weeks prior to the experiment added an average of 434.6 ± 21.7 mm^2^ (n = 34) of shell area over the course of the experiment. These oysters grew significantly slower under severe cycling hypoxia than under normoxia during the first two weeks of the experiment but not during the second two weeks, and ending shell areas were not affected (Table 7B, 7E and 7H, Fig 3B, 3E and 3H). Neither moderate cycling hypoxia nor cycling pH significantly affected growth rate of this cohort during any portion of the experiment (Table 7B, 7E and 7H, Fig 3B, 3E and 3H). Juveniles settled one week prior to the experiment grew an average of 337.8 ± 19.0 mm^2^ (n = 34) in shell area over the course of the entire experiment. These juveniles displayed a trend towards reduced shell area under severe cycling hypoxia in the first two weeks of the experiment (p = 0.056). There were no significant effects of moderate cycling hypoxia or cycling pH on growth (Fig 3C). During the second two week period, there were no significant effects of cycling hypoxia or pH on growth (Table 7F, Fig 3F). At the end of the experiment, oysters exposed to severe cycling hypoxia were 27% smaller than those exposed to normoxia, but there were no significant differences in size between oysters exposed to cycling pH or oysters exposed to moderate cycling hypoxia and those exposed to normoxia (Table 7I, Fig 3I). Constant moderate pH had no effects on growth during any time period in any age class.

**Table 7:** 2013 juvenile oyster growth experiment.

**A. 4 weeks post-settlement– 2.5 week Instantaneous Growth Rate**			
ANCOVA Source and Factor	df	F	p
Starting Shell Area	1, 25.97	31.63	**<0.001**
Treatment	6, 22.24	1.86	0.133
Contrasts	Df	t	p
Severe cycling hypoxia*Cycling pH Interaction	22	0.87	0.393
Severe cycling hypoxia vs. Normoxia	22	0.40	0.695
Severe cycling hypoxia vs. Moderate cycling hypoxia	22	0.28	0.780
Moderate cycling hypoxia vs. Normoxia	22	0.45	0.660
Cycling pH vs. Normocapnia	22	0.79	0.440
Constant moderate hypoxia vs. Cycling moderate hypoxia	22	2.15	**0.043**
Constant moderate hypoxia vs. Normoxia	22	2.14	**0.044**
Constant moderate pH vs. Normocapnia	22	0.79	0.436
Constant moderate pH vs. Cycling pH	22	0.03	0.974
**B. 2 weeks post-settlement– 2.5 week Instantaneous Growth Rate**			
ANCOVA Source and Factor	df	F	p
Starting Shell Area	1, 24.14	25.99	**<0.001**
Treatment	6, 23.03	6.80	**<0.001**
Contrasts	Df	t	P
Severe cycling hypoxia*Cycling pH Interaction	23	0.87	0.395
Severe cycling hypoxia vs. Normoxia	23	2.07	**0.050**
Severe cycling hypoxia vs. Moderate cycling hypoxia	23	0.41	0.684
Moderate cycling hypoxia vs. Normoxia	23	1.08	0.289
Cycling pH vs. Normocapnia	23	1.12	0.273
Constant moderate hypoxia vs. Cycling moderate hypoxia	23	4.19	**<0.001**
Constant moderate hypoxia vs. Normoxia	23	4.94	**<0.001**
Constant moderate pH vs. Normocapnia	23	0.30	0.771
Constant moderate pH vs. Cycling pH	23	0.00	0.999
**C. 1 week post-settlement– 2.5 week Shell Area**			
ANOVA Source and Factor	df	F	p
Treatment	6, 23	6.18	**<0.001**
Contrast	Df	t	p
Severe cycling hypoxia*Cycling pH Interaction	23	0.16	0.874
Severe cycling hypoxia vs. Normoxia	23	2.02	0.056
Severe cycling hypoxia vs. Moderate cycling hypoxia	23	0.47	0.644
Moderate cycling hypoxia vs. Normoxia	23	1.59	0.126
Cycling pH vs. Normocapnia	23	0.33	0.745
Constant moderate hypoxia vs. Cycling moderate hypoxia	23	3.83	**<0.001**
Constant moderate hypoxia vs. Normoxia	23	5.13	**<0.001**
Constant moderate pH vs. Normocapnia	23	1.25	0.224
Constant moderate pH vs. Cycling pH	23	1.3	0.206
**D. 4 weeks post settlement– 2.5–5.5 week Instantaneous Growth Rate**			
ANCOVA Source and Factor	df	F	p
Midpoint Shell Area	1, 22.64	10.35	**0.004**
Treatment	6, 22.88	2.52	0.051
Contrasts	df	t	p
Severe cycling hypoxia*Cycling pH Interaction	22	0.93	0.365
Severe cycling hypoxia vs. Normoxia	22	0.35	0.733
Severe cycling hypoxia vs. Moderate cycling hypoxia	22	0.10	0.919
Moderate cycling hypoxia vs. Normoxia	22	0.22	0.827
Cycling pH vs. Normocapnia	22	0.84	0.411
Constant moderate hypoxia vs. Cycling moderate hypoxia	22	3.11	**0.005**
Constant moderate hypoxia vs. Normoxia	22	2.87	**0.009**
Constant moderate pH vs. Normocapnia	22	0.29	0.774
Constant moderate pH vs. Cycling pH	22	0.59	0.562
**E. 2 weeks post-settlement– 2.5–5.5 week Instantaneous Growth Rate**			
ANCOVA Source and Factor	df	F	P
Midpoint Shell Area	1, 2	0.51	0.484
Treatment	6, 22.18	0.70	0.650
Contrasts	df	t	P
Severe cycling hypoxia*Cycling pH Interaction	22	0.33	0.744
Severe cycling hypoxia vs. Normoxia	22	0.68	0.502
Severe cycling hypoxia vs. Moderate cycling hypoxia	22	0.34	0.738
Moderate cycling hypoxia vs. Normoxia	22	0.04	0.965
Cycling pH vs. Normocapnia	22	0.08	0.939
Constant moderate hypoxia vs. Cycling moderate hypoxia	22	1.45	0.161
Constant moderate hypoxia vs. Normoxia	22	1.23	0.231
Constant moderate pH vs. Normocapnia	22	0.71	0.482
Constant moderate pH vs. Cycling pH	22	0.04	0.966
**F. 1 week post-settlement– 2.5–5.5 week Instantaneous Growth Rate**			
ANOVA Source and Factor	Df	F	P
Midpoint Shell Area	1, 22.31	0.00	0.986
Treatment	6, 22.20	0.77	0.604
Contrast	Df	t	P
Severe cycling hypoxia*Cycling pH Interaction	22	0.10	0.924
Severe cycling hypoxia vs. Normoxia	22	1.28	0.214
Severe cycling hypoxia vs. Moderate cycling hypoxia	22	0.72	0.481
Moderate cycling hypoxia vs. Normoxia	22	0.38	0.711
Cycling pH vs. Normocapnia	22	0.15	0.879
Constant moderate hypoxia vs. Cycling moderate hypoxia	22	1.82	0.083
Constant moderate hypoxia vs. Normoxia	22	1.67	0.109
Constant moderate pH vs. Normocapnia	22	0.21	0.834
Constant moderate pH vs. Cycling pH	22	0.24	0.811
**G. 4 weeks post-settlement–Endpoint shell area**			
ANOVA Source and Factor	df	F	p
Starting Shell Area	1, 24.529	1.98	0.172
Treatment	6, 22.376	5.35	**0.002**
Contrast	Df	t	P
Severe cycling hypoxia*Cycling pH Interaction	22	1.71	0.101
Severe cycling hypoxia vs. Normoxia	22	0.47	0.641
Severe cycling hypoxia vs. Moderate cycling hypoxia	22	0.98	0.340
Moderate cycling hypoxia vs. Normoxia	22	0.53	0.603
Cycling pH vs. Normocapnia	22	1.28	0.215
Constant moderate hypoxia vs. Cycling moderate hypoxia	22	3.51	**0.002**
Constant moderate hypoxia vs. Normoxia	22	2.97	**0.007**
Constant moderate pH vs. Normocapnia	22	1.29	0.212
Constant moderate pH vs. Cycling pH	22	0.77	0.447
**H. 2 weeks post-settlement–Endpoint shell area**			
ANOVA Source and Factor	df	F	P
Starting Shell Area	1, 24.673	0.04	0.840
Treatment	6, 22.221	5.86	**0.001**
Contrast	df	t	P
Severe cycling hypoxia*Cycling pH Interaction	22	0.47	0.642
Severe cycling hypoxia vs. Normoxia	22	1.49	0.151
Severe cycling hypoxia vs. Moderate cycling hypoxia	22	1.18	0.252
Moderate cycling hypoxia vs. Normoxia	22	0.13	0.898
Cycling pH vs. Normocapnia	22	0.27	0.793
Constant moderate hypoxia vs. Cycling moderate hypoxia	22	4.86	**<0.001**
Constant moderate hypoxia vs. Normoxia	22	4.59	**<0.001**
Constant moderate pH vs. Normocapnia	22	0.19	0.848
Constant moderate pH vs. Cycling pH	22	0.90	0.377
**I. 1 week post-settlement–Endpoint shell area**			
ANOVA Source and Factor	df	F	P
Treatment	6, 23	6.57	**<0.001**
Contrast	Df	t	P
Severe cycling hypoxia*Cycling pH Interaction	23	0.54	0.597
Severe cycling hypoxia vs. Normoxia	23	2.29	**0.031**
Severe cycling hypoxia vs. Moderate cycling hypoxia	23	0.6	0.554
Moderate cycling hypoxia vs. Normoxia	23	1.62	0.120
Cycling pH vs. Normocapnia	23	0.76	0.453
Constant moderate hypoxia vs. Cycling moderate hypoxia	23	3.90	**0.001**
Constant moderate hypoxia vs. Normoxia	23	5.32	**<0.001**
Constant moderate pH vs. Normocapnia	23	1.22	0.236
Constant moderate pH vs. Cycling pH	23	1.43	0.167

Randomized complete block design ANCOVA of instantaneous rate of growth in shell heights showing results from two age classes of juvenile oysters; (A) 4 weeks post-settlement, (B) 2 weeks post-settlement during first 18 days of experiment. (C) ANOVA for 18 day shell area of juvenile oysters from 1 week post-settlement oysters. (D, E, F) ANCOVA of instantaneous growth rate during second half of experiment in three age classes of oyster. (G, H), ANCOVA of shell area at the end of the 5.5-week laboratory exposure using starting shell areas as a covariate in the two older age classes of juvenile oyster. ANOVA of (I) ending oyster sizes from settlement 3:1 week post-settlement during 39 day experiment. Tests are considered significant at *a* = 0.05 and significant p values are bolded.

Growth of all three cohorts was significantly lower in the constant moderate hypoxia treatment than in either the cycling moderate hypoxia or normoxic control treatments during the first two weeks of the experiment (Table 7A, 7B and 7C, Fig 3A, 3B and 3C). In the second two weeks, constant moderate hypoxia did not affect growth rates in the oysters settled two weeks or one week before the experiment’s start, but did reduce growth rates in those oysters settled four weeks before the experiment (Table 7D, 7E and 7F, Fig 3D, 3E and 3F). All three age classes experienced a 25–33% reduction in shell area in the constant moderate hypoxia treatment (1.27 mg L^-1^ DO) at the end of the month-long course of the experiment (Table 7G, 7H and 7I, Fig 3G, 3H and 3I).

As a result of high spring and early summer precipitation, salinity during the 2014 growth experiment was at the extreme low end of the eastern oyster’s native range. This relatively low salinity water was also low in alkalinity; therefore, 2014 oysters may have experienced carbonate stress even in normocapnia treatments (Tables 5 and 8). Although the oysters were three weeks post-settlement at the start of the experiment and had been kept under well aerated conditions, they were still ≤1 mm in shell area when placed into experimental aquaria (Table 2).

**Table 8:** Mean ± SE, n, and range of calcite saturation states by treatment.

		Growth experiment year			
Treatment	DO & pH	2012	2013	2014	2015
Normoxia,	HDO/pH:	1.05 ± 0.001	1.81 ± 0.033	0.68 ± 0.018	1.01 ± 0.029
Normocapnia		17045	64	48	34
		(0.61–2.03)	(1.37–2.51)	(0.46–1.02)	(0.72–1.35)
Normoxia,	HDO/pH:	1.11 ± 0.001	1.94 ± 0.003	0.66 ± 0.003	0.87 ± 0.002
cycling pH		8071	2856	1099	3525
		(0.73–1.65)	(0.93–2.15)	(0.43–1.06)	(0.51–1.44)
	LDO/pH:	0.19 ± 0.000	0.18 ± 0.000	0.10 ± 0.000	0.05 ± 0.000
		2207	552	528	820
		(0.15–0.23)	(0.17–0.23)	(0.08–0.11)	(0.05–0.14)
Moderate	HDO/pH:		1.91 ± 0.002		
cycling			2694		
hypoxia,			(1.58–2.24)		
Normocapnia	LDO/pH:		2.18 ± 0.004		
			536		
			(2.02–2.46)		
Moderate	HDO/pH::	1.04 ± 0.001			
cycling		7800			
hypoxia,		(0.57–1.71)			
cycling pH	LDO/pH	0.194 ± 0.000			
		2202			
		(0.16–0.26)			
Severe cycling	HDO/pH:	1.00 ± 0.001	1.98 ± 0.001	0.71 ± 0.004	1.16 ± 0.034
hypoxia,		7800	2705	957	7
Normocapnia		(0.70–1.59)	(1.69–2.24)	(0.43–1.11)	(0.98–1.35)
	LDO/pH:	1.13 ± 0.001	2.14 ± 0.003	0.89 ± 0.007	0.94 ± 0.030
		2207	551	517	24
		(0.99–1.24)	(1.95–2.31)	(0.58–1.18)	(0.72–1.27)
Severe cycling	HDO/pH:			0.67 ± 0.003	
hypoxia,				961	
Moderate				(0.45–0.91)	
cycling pH	LDO/pH:			0.24 ± 0.001	
				528	
				(0.20–0.27)	
Severe cycling	HDO/pH:	1.04 ± 0.001	1.87 ± 0.003	0.68 ± 0.003	0.99 ± 0.002
hypoxia,		7578	2654	961	3459
cycling pH		(0.73–1.49)	(0.89–2.23)	(0.46–1.08)	(0.52–1.43)
	LDO/pH:	0.19 ± 0.000	0.18 ± 0.000	0.10 ± 0.000	0.05 ± 0.000
		2196	551	528	756
		(0.12–0.22)	(0.15–0.23)	(0.08–0.11)	(0.05–0.14)
Normoxia,	HDO/LpH:		0.43 ± 0.001		
Constant low			4402		
pH			(0.31–1.45)		
Constant	LDO/HpH:		1.84 ± 0.003		
moderate			4227		
hypoxia,			(1.29–2.76)		
Normocapnia					
Constant mild	LDO/HpH:			0.75 ± 0.002	
hypoxia,				2743	
Normocapnia				(0.49–1.06)	

Mean ± SE, n, and range of calcite saturation states by treatment for each experiment during the simulated day and night periods, high DO/pH and the low DO/pH periods. Calcite saturation state calculated using CO2SYS.XLS [55] from ten minute average LabVIEW data. Empty boxes are treatments which were not used during the experiment in that column.

Juvenile oysters grew an average of 11.1 ± 0.3 mm^2^ (n = 48) over the course of the two week 2014 experiment. Supplementing aquaria with a stock algal diet increased growth rates; however, the difference between oysters receiving supplemented and ambient food was on the order of 1 mm^2^ and there were no interactive effects of food availability with DO/pH treatment as had been expected (Table 9). Food level was therefore used as a blocking factor for further analysis in order to focus on DO and pH treatment effects.

**Table 9:** 2014 juvenile oyster growth experiment.

End Shell Area			
ANOVA Source and Factor	df	F	P
Food Treatment*DO/pH Treatment	5, 33	0.54	0.747
DO/pH Treatment	5, 33	3.68	**0.009**
Food Treatment	1, 33	4.13	**0.050**
Treatment	5,41	3.90	0.006
Contrast	df	t	P
Severe cycling hypoxia*Cycling pH Interaction	41	4.88	**0.033**
Severe cycling hypoxia vs. Normoxia under Normocapnia	41	9.01	**0.005**
Severe cycling hypoxia vs. Normoxia under Severe Cycling pH	41	1.33	0.256
Constant mild hypoxia vs. Normoxia under Normocapnia	41	11.79	**0.001**
Constant mild hypoxia vs. Cycling Severe hypoxia	41	0.06	0.803
Severe cycling pH vs. Normocapnia under Normoxia	41	5.78	**0.021**
Severe cycling pH vs. Normocapnia under Severe cycling hypoxia	41	0.02	0.898

Randomized complete block design 2-way ANOVA of DO/pH treatment by food treatment interaction (ANOVA 1) and ANOVA of shell area (ANOVA 2) from the end of the two week laboratory exposure. Tests are considered significant at *a* = 0.05 and significant p values are bolded.

**Fig 4:**
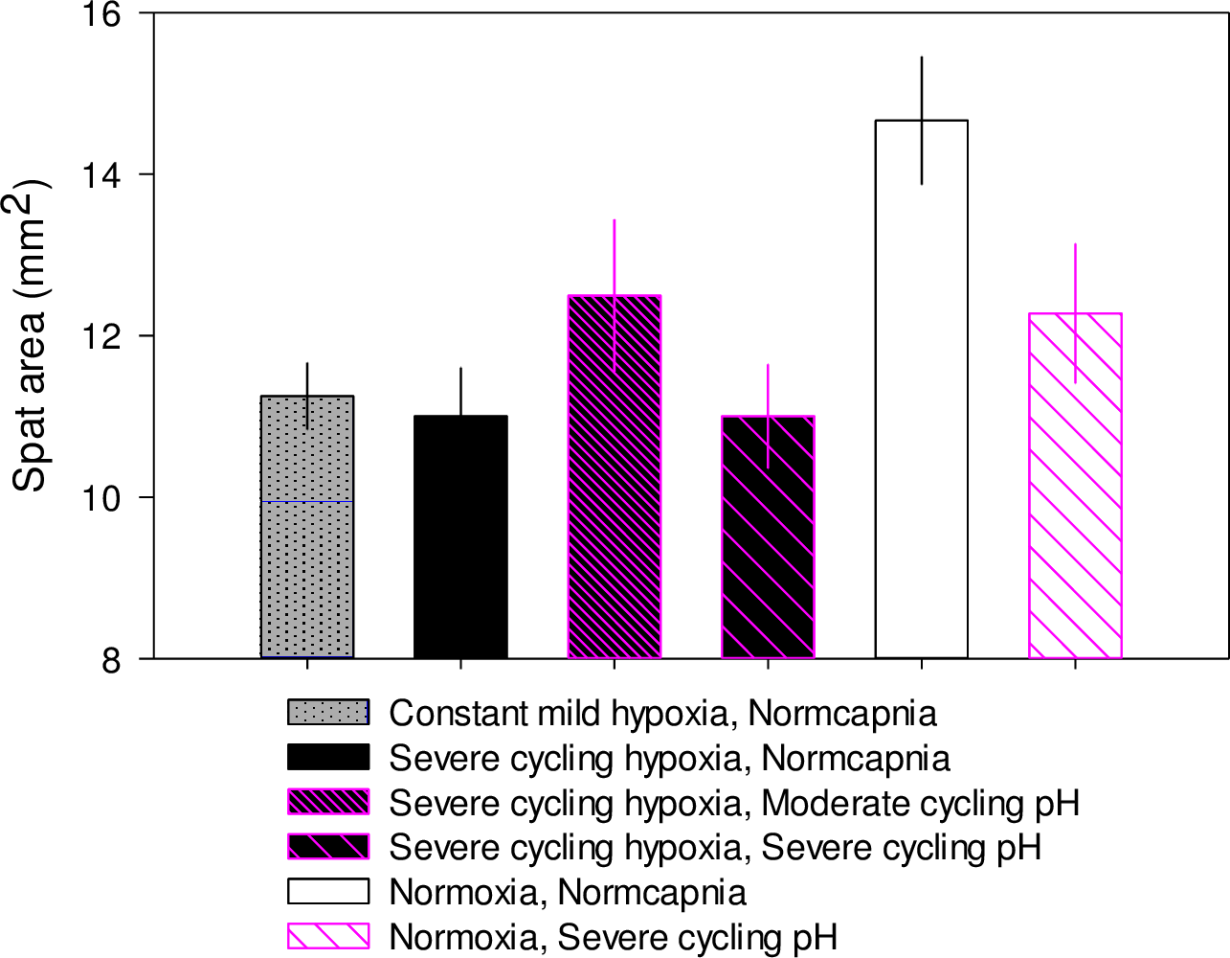
2014 juvenile growth experiment. Mean ± 1 SE shell area by treatment of juvenile oysters exposed to diel cycles 5–6 d wk^-1^ during a two-week laboratory experiment.

After two weeks of exposure to cycling conditions, there was a significant interaction between severe cycling hypoxia and cycling pH (Table 9, Fig 4). Under normoxic conditions, cycling pH reduced juvenile oyster shell area. Hypoxia reduced growth by a similar amount under both normocapnia and cycling pH. Constant mild hypoxia significantly reduced shell area by 12% compared to constant normoxia, a similar reduction to that of oysters exposed to cycling severe hypoxia at the experiment’s conclusion (Table 9, Fig 4).

**Fig 5:**
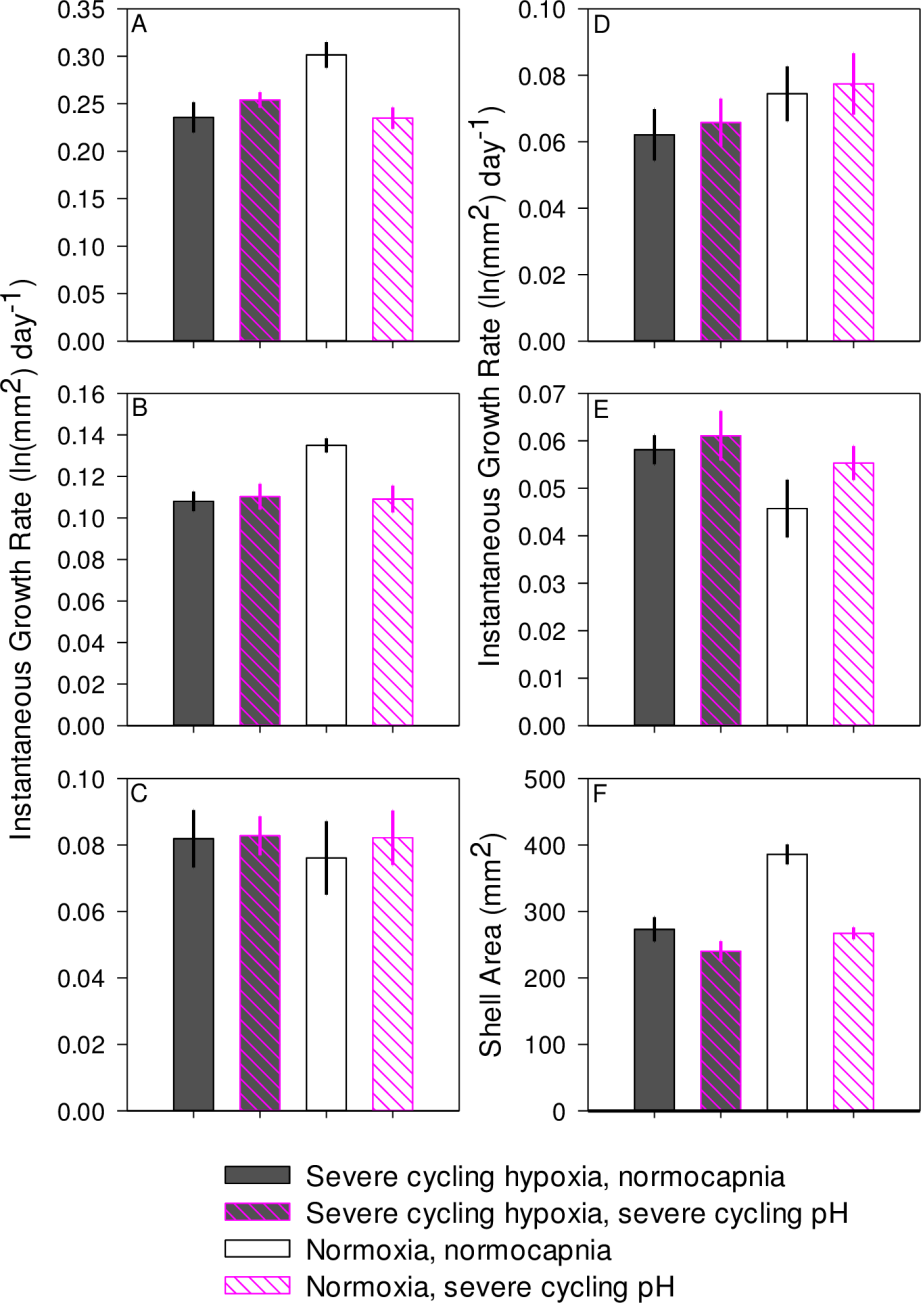
2015 juvenile growth experiment. Mean ± 1 SE instantaneous rate of growth by treatment of juvenile oysters exposed to diel cycles 7 d wk^-1^ during (A) first week, (B) second week, (C) third week, (D) fourth week, (E) fifth week, and (F) mean ± 1 SE shell area at the end of the five week laboratory exposure.

In 2015, four week post-settlement oyster shell sizes increased by an average of 283.0 ± 13.3 mm^2^ (n = 24) over the 5 week course of the experiment. Ω_calcite_ in controls was approximately one during this experiment—lower than in 2013, but higher than in 2014 (Table 8). During the first week of exposure to 2015 experimental treatments, there was an interactive effect of DO and pH on instantaneous growth rates (Table 10B, Fig 5A). Severe cycling hypoxia reduced growth rate under normocapnia by 28%, as did severe cycling pH under normoxia and the combination of the two stressors. This pattern continued through the second week, but in the third week, cycling pH had a significant stimulatory effect on growth, while in the fourth week cycling DO had a stimulatory effect on growth (Table 10C, 10D and 10E, Fig 5B, 5C and 5D). In the fifth week, there was a trend towards an interactive effect of cycling DO and cycling pH (Table 10F, Fig 5E), but in this case growth rates of oysters exposed to cycling DO or cycling pH were higher than those of the control treatment by 17–21%. At the conclusion of the laboratory treatment exposure, oysters exposed to cycling treatments were on average smaller than those exposed to control conditions in spite of the compensatory growth observed in the final weeks (Table 10G, Fig 5F).

**Table 10:** 2015 juvenile oyster growth experiment.

**A. Starting shell area**			
ANOVA Source and Factor	df	F	P
DO/pH Interaction	1, 15	3.02	0.103
DO Treatment	1, 15	1.93	0.186
pH Treatment	1, 15	0.56	0.468
**B. First Week Instantaneous Growth**			
ANCOVA Source and Factor	df	F	P
Starting Shell Area	1, 14.063	3.57	0.080
DO/pH Interaction	1, 16.016	9.57	**0.007**
DO Treatment	1, 15.079	2.24	0.155
pH Treatment	1, 14.34	5.15	**0.039**
Normoxia, normocapnia / Normoxia, cycling pH		3.84	**0.002**
Normoxia, normocapnia / Severe cycling DO, Normocapnia		3.31	**0.005**
Normoxia, normocapnia / Severe cycling DO, cycling pH		2.75	**0.016**
Normoxia, cycling pH / Severe cycling DO, normocapnia		0.23	0.821
Normoxia, cycling pH / Severe cycling DO, cycling pH		1.11	0.284
Severe cycling DO, normocapnia / Severe cycling DO, cycling pH		0.82	0.425
**C. Second Week Instantaneous Growth**			
ANCOVA Source and Factor	df	F	P
One Week Shell Area	1, 16.958	0.20	0.657
DO/pH Interaction	1, 14.045	4.67	**0.049**
DO Treatment	1, 14.486	4.63	**0.049**
pH Treatment	1, 14.002	5.82	**0.030**
Normoxia, normocapnia / Normoxia, cycling pH		3.39	**0.004**
Normoxia, normocapnia / Severe cycling DO, Normocapnia		3.37	**0.005**
Normoxia, normocapnia / Severe cycling DO, cycling pH		3.30	**0.005**
Normoxia, cycling pH / Severe cycling DO, normocapnia		0.11	0.915
Normoxia, cycling pH / Severe cycling DO, cycling pH		0.04	0.968
Severe cycling DO, normocapnia / Severe cycling DO, cycling pH		0.07	0.948
**D. Third Week Instantaneous Growth**			
ANCOVA Source and Factor	df	F	P
Two Week Shell Area	1, 14.278	24.75	**<0.001**
DO/pH Interaction	1, 17.255	2.24	0.152
DO Treatment	1, 16.621	1.72	0.208
pH Treatment	1, 18.903	7.19	**0.015**
**E. Fourth Week Instantaneous Growth**			
ANCOVA Source and Factor	df	F	P
Three Week Shell Area	1, 17.32	2.73	0.116
DO/pH Interaction	1, 14.969	1.20	0.290
DO Treatment	1, 14.515	6.42	**0.023**
pH Treatment	1, 16.547	1.77	0.201
**F. Fifth Week Instantaneous Growth**			
ANCOVA Source and Factor	df	F	P
Four Week Shell Area	1, 16.953	45.15	**<0.001**
DO/pH Interaction	1, 18.024	3.95	0.062
DO Treatment	1, 18.579	0.05	0.822
pH Treatment	1, 17.022	3.63	0.074
Normoxia, normocapnia / Normoxia, cycling pH		3.12	**0.008**
Normoxia, normocapnia / Severe cycling DO, Normocapnia		2.49	**0.026**
Normoxia, normocapnia / Severe cycling DO, cycling pH		2.72	**0.017**
Normoxia, cycling pH / Severe cycling DO, normocapnia		1.12	0.281
Normoxia, cycling pH / Severe cycling DO, cycling pH		0.01	0.995
Severe cycling DO, normocapnia / Severe cycling DO, cycling pH		1.07	0.303
**G. Endpoint Shell Area**			
ANCOVA Source and Factor	df	F	P
Starting Shell Area	1, 9.579	1.91	0.198
DO/pH Interaction	1, 17.685	30.41	**<0.001**
DO Treatment	1, 16.156	64.41	**<0.001**
pH Treatment	1, 14.711	59.24	**<0.001**
Normoxia, normocapnia / Normoxia, cycling pH		9.50	**<0.001**
Normoxia, normocapnia / Severe cycling DO, Normocapnia		9.19	**<0.001**
Normoxia, normocapnia / Severe cycling DO, cycling pH		11.43	**<0.001**
Normoxia, cycling pH / Severe cycling DO, normocapnia		0.65	0.526
Normoxia, cycling pH / Severe cycling DO, cycling pH		1.86	0.084
Severe cycling DO, normocapnia / Severe cycling DO, cycling pH		1.09	0.296

(A) Randomized complete block design 2-way ANOVA of DO/pH treatment on starting shell area of oysters. (B-F) Randomized complete block design 2-way ANCOVA of DO/pH treatment effects on instantaneous rate of growth of juvenile oysters during each of the five weeks of the experiment. (G) Randomized complete block design 2-way ANOVA of DO/pH treatments on shell area at the end of the 5-week experimental period. Tests are considered significant at *a* = 0.05 and significant p values are bolded.

## Discussion

Our results indicate that exposure to brief repeated periods of hypoxia and low pH or to prolonged moderate hypoxia, can reduce instantaneous growth rates of juvenile oysters. The presence and magnitude of these transient effects varied among experiments and may have been modulated by inter-annual variation in salinity, ambient Ω_calcite_, oyster age, and initial size. Our results also indicate that juvenile oysters can often acclimate to cycling conditions or prolonged moderate hypoxia, and exposed oysters sometimes exhibit compensatory growth. By the end of lab experiments or post-experiment field deployments, oysters were frequently the same size regardless of experimental exposure to cycling hypoxia and/or low pH and initial negative effects of experiment treatments on growth rates. Although oyster mortality in the absence of predators was not affected by cycling or constant stressors we tested, temporary growth reductions could increase susceptibility to predation. Additionally, changes in energy allocation that allow growth rates to be preserved could alter reproduction or the ecosystem services provided by oysters. In the discussion below, we assume effects resulting from pH treatments were actually caused by the effect of our CO_2_ additions on Ω_calcite_ but generally refer to pH effects because this was the parameter manipulated in our experiments.

### Hypoxia Effects

Juvenile oyster growth was reduced under severe diel-cycling hypoxia (0.5 mg L^-1^) in the initial weeks of experiments in most years and age classes of oysters. The resulting difference in size between oysters exposed to severe diel-cycling hypoxia and the normoxia/normocapnia controls where effects were statistically significant ranged from about 30% in 2012 to 37% in 2015. The less consistently negative effect of severe cycling hypoxia during the first weeks of the 2013 experiment than in other years, may have reflected the lower mean temperature, which likely reduced metabolic demands [59]. The magnitude of the difference between the control oysters and those exposed to severe diel-cycling hypoxia in 2013 decreased with increasing initial size of cohorts. Oysters in the 2012 experiment were a similar age to and much larger than the oldest cohort of 2013 oysters, but showed effects of hypoxia similar to those in the smallest oysters in 2013, suggesting that initial size alone did not fully explain among- and within-year variability in responses.

Moderate cycling hypoxia (1.7 mg L^-1^ in 2012 and 1.3 mg L^-1^ in 2013) had no overall effect on oyster shell area; although it did sometimes reduce instantaneous growth rates of oysters. The absence of moderate cycling hypoxia effects even when there were negative effects of severe cycling hypoxia indicate that there may be a threshold of hypoxia somewhere between 0.5 and 1.5 mg L^-1^ at which oyster growth is affected, similar to the potential threshold of hypoxia for disease effects [50] or thresholds for behavioral responses to low DO [44,60,61]. Variation in sensitivity among 2013 juvenile oyster age classes indicates that the threshold for hypoxia effects likely varies with oyster age and size.

Our experiments also indicate that duration and severity of hypoxia exposure can influence the magnitude of initial effects on growth. In 2014, constant mild hypoxia (2.0 mg L^-1^) and brief periods of severe hypoxia (to 0.5 mg L^-1^ DO 5–6 d wk^-1^) each reduced juvenile growth rates measured following two weeks of exposure by similar amounts. However, exposure to a somewhat lower level of constant moderate hypoxia (1.3 mg L^-1^) in 2013 reduced juvenile growth far more than did exposure to severe cycling hypoxia (0.5 mg L^-1^) in that year. Cycling conditions provide periods of respite at high oxygen which can allow for compensatory feeding and the repayment of oxygen debt [23,52,54,62], potentially allowing oysters to grow more quickly under cycling conditions than constant hypoxic conditions even when minimum DO concentrations to which oysters are exposed are much lower.

### pH Treatment Effects and DO by pH Interactions

Organisms will rarely be exposed to a single stressor in isolation [63–65]. Low salinity, corresponding low alkalinity, and resulting low calcite saturation would be expected to increase the susceptibility of oysters to the harmful effects of hypercapnia treatments and perhaps increase susceptibility to hypoxia because of increased energetic costs of shell production [28,66]. In addition to effects on alkalinity, low salinity can reduce the rate of nutrient assimilation in oysters [28], and thus the energy available to overcome other stressors.

Cycling pH affected growth of oysters during three of our four experiments, but the direction of effects and interaction with DO treatments varied among years and we sometimes saw stimulatory effects of cycling pH. In 2013, under the highest salinity and alkalinity, and thus highest ambient Ω_calcite_, there was no negative effect of the pH cycle alone. This was also the only condition under which a constant moderate hypercapnia (~7.35 pH) treatment was tested, and no effect of this treatment was observed. Exposure to cycling pH did negatively affect growth during the first two weeks of the 2014 and 2015 experiments which had the lowest Ω_calcite_ minima for the cycling pH treatments. Furthermore, in 2012 when minimum Ω_calcite_ was 0.18 and 2015 when minimum Ω_calcite_ was 0.05, after several weeks of exposure, oysters in some cycling pH treatments grew faster than controls, possibly due to stimulated feeding under low pH conditions [52,54].

Calculations of Ω_calcite_ (Table 8) indicated that under low salinity and alkalinity conditions of 2014, all juvenile oysters, even those not intentionally exposed to pH stress, were exposed to severe carbonate stress. In 2015, at higher relative ambient Ω_calcite_ levels, more severe pH treatments resulted in low pH Ω_calcite_ levels similar to those in 2014. Although we cannot be certain whether results were due to ambient environmental conditions or to treatments that resulted in lower Ω_calcite_ during the hypercapnia phases of cycling pH treatments than in 2012 and 2013, oyster growth was negatively impacted by *both* cycling hypoxia and cycling pH in 2014 and 2015.

The effect of pH varied among DO treatments in several experiments. In the 2014 and 2015 experiments, the combination of cycling pH and severe cycling hypoxia during the first two weeks resulted in growth reductions equivalent to those of severe cycling hypoxia or cycling pH independently. In contrast, exposure to both cycles in the second half of the 2012 experiment resulted in higher growth rates than in oysters exposed to either cycle independently while in the second half of the 2015 experiment cycling pH resulted in more rapid growth only under normoxic conditions. In a similar experiment, juvenile tubeworms, *Hydroides elegans*, showed reduced expression of calcification related proteins under either hypoxia or hypercapnia but protein expression returned to control levels when exposed to both stressors simultaneously [67].

### Acclimation and Compensation

Oysters acclimated to treatment conditions, or compensated for early reductions in growth under hypoxic exposure or combined exposure to cycling hypoxia and pH, in all experiments more than two weeks in duration except for four-week post-settlement cohort in 2013. Acclimation, in this case, is defined as declining severity of effects over the course of an experiment, while compensation is defined as stimulatory effects later in exposure that ultimately eliminate initial negative effects of stressors. Acclimation and compensation appeared to include both short-term behavioral or physiological responses related to hypoxia and low pH exposure, as well as more persistent changes in oysters exposed to hypoxia. Bayne [68] demonstrated that other oyster species can modify feeding behaviors to maintain necessary energy uptake rates under fluctuating environmental conditions. The combination of compensatory feeding during high oxygen portions of the cycle with increased feeding under low pH as seen in older oysters [52,54] may have allowed juvenile oysters exposed to cycles of both DO and pH to grow as quickly as oysters exposed to non-cycling conditions. Longer term compensatory growth may have resulted from more persistent morphological changes such as increased gill size. Under hypoxic conditions, oysters have been shown to develop larger gill area to improve ventilation efficiency [68]. During the nine month respite from laboratory cycling conditions following the 2012 experiment, juvenile oysters exhibited compensatory growth, which resulted in similar sized oysters among treatments. These results indicate lingering effects of treatment exposures on energy allocation, physiological responses or morphological adaptations in previously hypoxia-exposed oysters.

In the 2012 growth experiment, oysters compensated for moderate cycling hypoxia exposure as well as exposure to co-varying cycles of severe hypoxia but only in the presence of co-varying cycles of pH. In this experiment, early exposure to moderate cycling hypoxia resulted in reduced growth. Growth rates during the second two week period were, however, similar to those of the controls and, by the end of the experiment, shell areas were not significantly different from those of oysters never exposed to hypoxia, indicating that oysters had compensated for early reductions in growth. The middle and youngest age classes of juvenile oysters in 2013 acclimated to early negative effects of severe cycling hypoxia. The salinity and alkalinity in 2013 were much higher than in 2012, which may have allowed for acclimation to more severe cycling conditions than in the previous year due to a lower energetic cost of calcification. Two of the three age classes of juvenile oysters in the 2013 experiment acclimated to constant moderate hypoxia. In 2015, at salinity and alkalinity levels between those of the previous experiments, oysters acclimated to cycling conditions during the third week of exposure, and, by the fifth week, were growing more quickly than those exposed to normoxia. These results suggest that oysters are well adapted to cycling conditions and exhibit plasticity that can allow them to overcome exposure to negative conditions [68,69].

Shifts in energy allocation between shell growth and other metabolic costs may have contributed to the ability of oysters to acclimate to hypoxia or hypercapnia or to exhibit compensatory growth. While some research indicates that shell growth is a prioritized activity in oysters (*C*. *gigas*) [28], other research has shown that freshwater clams, *Anodonta piscinalis* (L., 1758), preferentially reduce energy allocation to shell growth before sacrificing maintenance or reproduction [70]. Energy allocation may also change with life stage: for instance, younger animals may prioritize growth, while an older animal may preferentially put energy towards reproduction [71]; on the other hand, younger animals may be more energy limited due to more rapid growth rates and smaller energy reserves. This might leave less energy to allocate to reproduction in spite of being similar in size to oysters not previously exposed to hypoxia [72], potentially reducing reproduction effort whether or not cycling conditions abate. Although our experiments were on juvenile oysters, shifts in allocation could affect the likelihood of reproduction in the following summer.

### Implications

Reduced oyster size caused by exposure to diel-cycling hypoxia and pH may diminish important ecosystem services including provision of oyster bar habitat and water filtration [39], may reduce fecundity as smaller oysters produce fewer eggs or sperm each season [73], and may increase susceptibility to predation [37,74]. However, our results indicate that, at least under some environmental conditions, juvenile oysters have an ability to acclimate to, and ultimately compensate for, the negative effects of hypoxia on growth as well as an ability under some circumstances to withstand exposure to co-varying cycling hypoxia as low as 0.5 mg L^-1^ and pH as low as 7.0 without reductions in growth. Nevertheless, increased disease loads in adult oysters under severe cycling hypoxia [6,50]may have important effects on population viability. Adult oysters may also have less capacity for compensatory growth when exposed to cycling hypoxia than the juveniles tested here [6].

Under global climate change, the Chesapeake Bay region is predicted to become warmer and drier [75,76]. While the small range of temperatures tested did not appear to interact with cycling conditions in this experiment, higher temperature might both increase the severity of hypoxic events [76,77], act as an additional estuarine stressor [78–80], and increase oxygen demand [45]. Drier conditions will increase salinity in some areas, resulting in higher alkalinity and increasing calcite availability, which might, given the results here, reduce effects of cycling conditions on growth in oysters, but also increase the risk of disease [81].

It would be interesting to look at extended periods of recovery after laboratory exposure to diel-cycling conditions to determine how long growth rates in oysters previously exposed to severe cycling hypoxia might remain stimulated. Potential latent effects of previous exposure to diel-cycling conditions on fecundity are also worthy of further investigation. Finally, we do not know how these cycling conditions might affect larval oysters and oyster recruitment. Younger individuals than those tested may be more susceptible to conditions that affect energy availability, and conditions more severe than those tested could possibly have more consistent negative effects. Although our experiments highlight the ability of juvenile oysters to acclimate to, or compensate for, exposure to cycling conditions, there may still be mechanisms and conditions that result in negative population-level effects.

## Supporting Information

S1 Table 2012 Oyster growth raw data. (XLSX)

S2 Table 2013 Oyster growth raw data. (XLSX)

S3 Table 2014 Oyster growth raw data. (XLSX)

S4 Table 2015 Oyster growth raw data. (XLSX)
